# Novel cardiac abnormalities observed in CAH patients with tenascin-X haploinsufficiency

**DOI:** 10.3389/fendo.2026.1797669

**Published:** 2026-05-15

**Authors:** Andrea Sappl, Annie M. Sriramachandran, Christian Lottspeich, Katharina Vill, Monika Morak, Ann-Christin Welp, Orsela Dervishi, Martin Bidlingmaier, Sonja Kunz, Nicole Reisch

**Affiliations:** Medizinische Klinik IV, LMU Klinikum, Ludwig-Maximilians-Universität München, Munich, Germany

**Keywords:** CAH-X syndrome, chimeric *TNXA/TNXB* gene, congenital adrenal hyperplasia, Ehlers-Danlos syndrome, tenascin X haploinsufficiency

## Abstract

**Background:**

Defects in both *CYP21A2* and *TNXB* genes cause congenital adrenal hyperplasia combined with hypermobility-type Ehlers–Danlos syndrome (EDS), which has been named CAH-X syndrome.

**Objective:**

This study aimed to determine the frequency of CAH-X within the Munich cohort of CAH patients (*n* = 155: salt wasting = 94, simple virilizing = 44, non-classical = 12, gene carrier = 4, 11β-deficiency = 1) and assess its clinical implications by thorough clinical characterization of the cohort. In addition, sTNXB protein levels were linked to clinical observations to alleviate potential consequences of mutations such as cardiovascular or joint problems.

**Design/setup:**

PCR screening for the presence of disease-causing variants in *TNXB* as well as in *CYP21A2* (*n* = 155) was conducted; simultaneously, with written consent from the patients (*n* = 76), clinical examinations for joint or skin abnormalities, muscle strength, and neurological functions were performed. CAH-X-positive patients were matched to two control CAH patients according to age and body mass index (BMI). sTNXB protein level estimation, muscle ultrasound, and echocardiography examinations were made on selected CAH-X and CAH patients.

**Outcome/conclusion:**

In our cohort, only 5% carry the CAH-X CH1 chimera. Besides EDS-related clinical symptoms, increased muscle echogenicity compared to unaffected matched controls, particularly in the legs, and cardiac abnormalities that have not been observed in other cohorts and are associated with underlying CAH-X, such as persistent truncus arteriosus and relaxation disorder, were observed. They highlight the importance of genetic as well as clinical screening and regular follow-up examinations for the CAH-X syndrome.

## 1 Introduction

Genes encoding 21-hydroxylase, *CYP21A2*, and an extracellular matrix protein tenascin X, *TNXB*, are located on the short arm of chromosome 6 within the major histocompatibility complex (MHC) loci (1). Defects in both *CYP21A2* and *TNXB* genes can cause congenital adrenal hyperplasia combined with hypermobility-type Ehlers–Danlos syndrome-like clinical symptoms (EDS), which has been named the CAH-X syndrome. Combined defects happen because both genes are located in the following tandem order: *RP1–C4A–CYP21P–XA–RP2–C4B–CYP21–TNXB*, forming the RCCX module within the MHC loci (2, 3). The *TNXB* gene overlaps with the *CREBLL1* and *CYP21A2* genes at the 5′ and 3′ ends, respectively. The high degree of polymorphism within the human leukocyte antigen (HLA) genes and the presence of homologous pseudogenes render this RCCX module more vulnerable to unlicensed homologous recombination (HR) events, resulting in genomic alterations such as copy number variations in genes, deletions, down- or upregulation of genes, duplications, and gene conversions. The CAH-X syndrome is reported to occur in 7%–15% of CAH patients (4, 5).

Tenascin X (TN-X or TNXB) is the largest glycoprotein in the tenascin family of proteins. It has an N-terminal ‘head piece’ responsible for polymerization of monomers forming a multi-branched ‘brachion’ structure, followed by a stretch of EGF-like repeats, a stretch of fibronectin type III (Fn III) repeats, and a C-terminal highly conserved fibrinogen-like domain (FBG-domain) (6). Point mutations that abolish TNXB function have been implicated in classical-like Ehlers–Danlos syndrome (clEDS). clEDS is a heterogeneous group of disorders with primary symptoms such as joint hypermobility, frequent dislocations, high susceptibility to skin injury, delayed wound healing, an increased occurrence of uterine and vaginal prolapse in women, and cardiovascular abnormalities (1). Besides its architectural function in tissue integrity, it stimulates vascular endothelial cell proliferation by simultaneous interaction via its Fn III repeats with vascular endothelial growth factor B (VEGF-B) and VEGF receptor 1 (VEGFR-1) (7, 8). Low levels of TNXB have been reported in five different types of cancers to cause an upregulation of TGF-β1-regulated genes (9, 10). Using mouse models, TNXB deficiency has been demonstrated to promote tumour metastasis (11). TNXB function in inhibiting TGF-β1 signaling has been demonstrated to be critical in regulating vascular smooth muscle cells (VSMCs) upon injury (12).

In 2013, a monoallelic appearance of *TNXA/TNXB* chimeric genes in approximately 5%–10% of CAH patients combined with various symptoms of hypermobility was first described (1). To date, three types of CYP21A2-TNXA/TNXB chimeras have been reported. CAH-X chimera 1 (CAH-X CH1) is the cause of tenascin-X haploinsufficiency and features a 120-bp deletion within the coding sequence of *TNXB*. CAH-X chimera 2 (CAH-X CH2) is associated with severe EDS symptoms and the missense variant p.Cys4058Trp. CAH-X chimera 3 (CAH-X CH3) contains three missense variants: p.Ser4175Asn, p.Asp4172Asn, and p.Arg4073His. Meanwhile, based on *in silico* analysis, CAH-X CH2 and CH3 are hypothesized to cause structural changes in the TNXB protein; CAH-X CH1-encoded protein levels have been reported to be reduced in dermal tissue (4). CAH-X CH1 chimera occurs more frequently in CAH patients (13). Besides these, other *TNXB* variants have also been identified in patients (14).

This study aimed to determine the frequency of CAH-X chimera within the Munich cohort of patients with CAH and assess their clinical implications by correlating the levels of serum TNXB protein with the clinical observations made in our cohort to alleviate potential consequences of *TNXB* missense variants such as cardiovascular or joint problems.

## 2 Materials and methods

### 2.1 Characteristics of the participants

Clinical examinations and PCR screening for CAH-X chimera were performed on 155 patients (above 18 years of age) at the Department of Endocrinology, Ziemssenstraße 1, Munich. All patients had a confirmed diagnosis of either salt wasting, simple virilizing, or a non-classical form of CAH based on both clinical observations and genetic phenotyping. All patients received glucocorticoid treatment (mean dose = 23.3 mg/day, dose range between 10 and 35 mg/day) at the time of examination. A written consent was obtained in advance.

### 2.2 Screening for the *TNXA/TNXB* chimera

A total of 155 patients with CAH were genotyped for the presence of *TNXA/TNXB* -chimera, most commonly caused by homologous recombination due to the high degree of polymorphism in this gene locus (15). Multiplex ligation-dependent probe amplification (MLPA) screening was performed on patient blood samples using the SALSA MLPA P155-B1 EDS Kit (MRC Holland, Amsterdam, Netherlands) as per the manufacturer’s instructions. Data were analyzed using the Coffalyser 9.4 software. PCR screening was performed in duplicates. Long-range PCRs covering *CYP21A2* and exons 32–44 of *TNXB* were performed on these seven patient samples. Classic CYP779f and Tena32F primers were used to generate an 8.5-kb-long amplicon (16). The amplification reaction was performed using the Expand Long Range dNTPack (Roche, Cat No. 4829034001, Basel, Switzerland). Prior to sequencing, the amplicons were purified using GenUP™ Exo SAP kit (biotechrabbit, Cat No. BR0701802, Berlin, Germany). Within the amplicon, a 2.4-kb region spanning exons 32 to 35 was sequenced to confirm the 120-bp deletion within the *TNXB* gene using primers as published previously (1). Sequencing was carried out in cooperation with Medizinisch Genetisches Zentrum Munich (MGZ).

### 2.3 Measurement of serum TNXB levels

Serum TNXB protein concentrations were measured in 35 patients, since previous studies implicated its function in maintaining the stability of elastic fibers in the extracellular matrix (15). ELISA assays were performed in patient blood samples using the ELISA kit (Cloud-Clone Corporation, Cat No. SEB935Hu) according to the manufacturer’s instructions. In brief, 100 µL of each blood sample was incubated at 37 °C using a 96-well microplate (Corning^®^ 96 Well Half-Area Microplate, Sigma, CLS3695-100EA) for 1 h. Assay diluent A (100 µL) was added to each sample and incubated further for an hour. Samples were washed thrice with 250 µL of wash buffer (PBS containing 0.05% Tween 20). A total of 100 µL of assay diluent B was added to each sample and incubated at 37 °C for 30 min, followed by the addition of 90 µL of substrate solution and further incubation for 10–20 min at 37 °C. The reactions were stopped by adding 50 µL of Stop Solution (2 N sulfuric acid). The optical density of each well was measured at a 450-nm wavelength using a microplate reader (FLUOstar^®^ Omega, BMG LABTECH).

### 2.4 Clinical examination

Genetically tested patients were examined for clinical findings associated with the Ehlers–Danlos syndrome. Patients were asked to fill in a questionnaire regarding their lifestyle, pre-existing conditions, frequency of joint and muscle pain, headaches, and urethral and gastrointestinal complaints (17). Body composition was measured by the body impedance analysis to assess fat and muscle composition in patients. Patients were asked to repeat certain movements, like flexion of the hip or arms, in a certain rhythm (mostly 40 bpm) up to 60 times and determine the FI-2 upper extremity score to assess the weight of each patient. Afterwards, subjective exertion was queried by using the Borg CR10 scale.

#### 2.4.1 Neurological examination

Following the standard procedure of the Friedrich-Baur-Institute for Neurology, four points were examined: deltoid, brachioradialis, thigh, and tibia. Neurologic examinations included the following: i) the Rydel–Seiffer test, to assess sense of vibration using a low-frequency (128 Hz) tuning fork on the inner ankle and wrist of the participants, followed by recording the intersection position (0 to 8) on the fork when the participant no longer senses vibrations; ii) the finger-nose test, where the participants were asked to alternatingly touch their nose and the examiner’s finger while the examiner moves own finger in different directions and the number of participants’ successful events in 30 s is recorded; iii) the Romberg test, to assess sense of balance, where the participants with their eyes closed and arms stretched forward were asked to stand still for 60 s; and iv) a 30-s chair stand test, to assess lower limb strength and endurance. Pain sensitivity was measured using an algometer. Pressure is applied at a constant rate of 1 kg/cm^2^ per second. The pressure at which the participant verbally indicates pain or discomfort is recorded as the pain pressure threshold (PPT). Pain sensitivity was examined at the tibia, thighs, deltoid, and brachioradialis muscles (18).

#### 2.4.2 Joint hypermobility and skeletal examination

The Beighton and Brighton scores were raised to detect joint hypermobility. For the Beighton 9-point scale, patients were examined for their ability to i) passively dorsiflex the fifth metacarpophalangeal joint beyond 90°, ii) oppose their thumb to the volar aspect of the ipsilateral forearm, iii) extend their knees beyond 10°, iv) extend their elbow beyond 10°, and v) bend forward and place the palms of their hands on the floor without bending the knee. For each positive result, 1 point was given, with 9 being the total score. When patients scored 4 or more points, the Beighton scale was considered to be positive (19). The Brighton score is considered pathologic with either i) two major criteria being positive, ii) one major and two minor criteria being positive, or ii) four minor criteria being positive. A positive Beighton score and pain in four or more joints were considered major criteria, while minor criteria included pain in up to three joints, back pain, or spondylolisthesis (20).

#### 2.4.3 Muscular examination

Muscle ultrasound examinations were performed using a GE Logic E9 ultrasound machine (General Electric, Munich, Germany) (21). For the classification of the observed phenotype, a modified Heckmatt scale was employed (22). It divides the results into four gradations: grade 1 refers to normal echogenicity of the examined muscle, grade 2 refers to increased muscle echogenicity with normal bone reflection, grade 3 refers to increased muscle echogenicity and reduced bone reflection, and grade 4 refers to a stark increase in muscle echogenicity and complete loss of bone reflection. Examinations were performed on five CAH-X-affected patients and the corresponding five CAH control patients without CAH-X syndrome. The muscles examined were the quadriceps, adductor muscles, the gastrocnemius, abdominal muscles, muscles of the back, deltoid, and finger extensors, as well as the median nerve. The physical strength of six muscle groups—shoulder abductors, elbow flexors, wrist extensors, hip flexors, knee extensors, and foot dorsiflexors—was evaluated using the Medical Research Council (MRC) scale: 5 points for full strength, 0 points for no muscle contraction at all, and 1 to 4 reflecting increasing muscle strength (23). Additionally, arm strength was assessed using a dynamometer to measure the force of muscle contraction with a handheld device.

#### 2.4.4 Dermatological examination

Questionnaires provided for the participants included the frequency of skin injury, skin scarring, formation of striae, and detectable scars. The skin was additionally examined for striae. Skin hyperextensibility was measured at the triceps. A skinfold caliper was used to measure subcutaneous fat at the triceps and subscapular areas (24).

#### 2.4.5 Cardiovascular examination

Cardiovascular fitness was examined for 15 participants (5 CAH-X and corresponding 10 matched CAH control patients) by performing step tests where the participant is asked to step on and off a platform of a specific height at a set pace for a 5-min period. After 5 min, heart rate was measured during recovery. This reflects the efficiency of both the cardiovascular and respiratory systems in delivering oxygen to muscles during exercise. Then, transthoracic echocardiographic examination was performed on all five CAH-X patients using high-quality ultrasound systems (GE Health Care Vivid 7, Phillips iE 33). The parameters used in the examination were in accordance with the guidelines of the American Society of Echocardiography (25). Special attention was given to detecting i) valve abnormalities or insufficiencies, ii) changes in the aorta such as aneurysms and dissections, and iii) ventricular changes and atrial septal defects.

### 2.5 Statistical analysis

Data were collected using MS Excel and analyzed using the SPSS program for Windows. Conditional logistic regression was used to account for the 2:1 (control:test subjects) matching correlation analysis. Non-normal distribution was assumed. The affected and non-affected patient tendencies were described to compare the two groups. Statistical significance was determined using both paired and unpaired *t*-tests to assess differences within each group and across all groups. Graphs were generated using GraphPad Prism.

## 3 Results

### 3.1 Genetic screening for *TNXA/TNXB* chimera in patients with CAH

**Figure 1:**
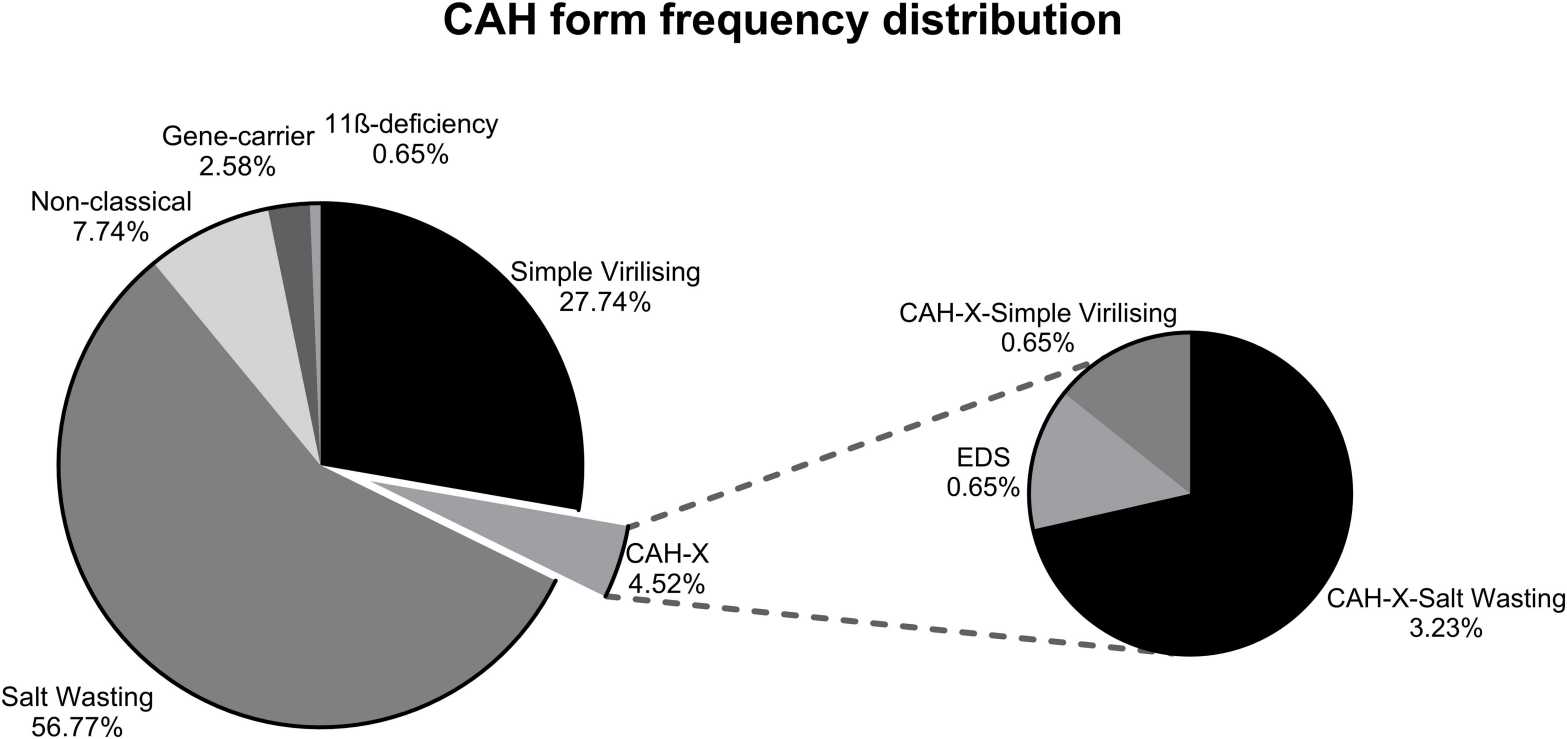
Frequency distribution of CAH forms in CAH-X and CAH patients in our study cohort. Genotyping was performed on 155 patients to determine the CAH form. These patients were also genotyped for the CAH-X CH1 chimera. A total of 7/153 tested positive, carrying the CAH-X CH1 chimera. The graph reflects the percentage of different forms of CAH observed in both CAH and CAH-X patient cohorts. One CAH patient had 11β-hydroxylase deficiency instead of 21-OH.

PCR screening was performed in patients with CAH (*n* = 155) for the presence of the CAH-X CH1 chimera. The reactions were performed in duplicates. Seven patients reproducibly tested positive in both PCR runs, indicating an intact *TNXB* gene as well as the presence of a highly homologous pseudogene. Two affected patients were related. Sequencing exons 32–35 in the five affected patients (CAH-X, case) revealed a 120-bp deletion within the *TNXB* gene, characteristic of the CAH-X CH1 chimera. Furthermore, a few previously known silent mutations were detected. In our CAH patient cohort, less than 5% carried the CAH-X CH1 chimera. A monoallelic *TNXA/TNXB* chimera was observed in six patients, and a biallelic *TNXA/TNXB* chimera was observed in a single patient, diagnosed previously with EDS (**Figure 1**). As the resulting tenascin-X haploinsufficiency in all patients is caused by a large gene deletion on one allele, all the matched CAH patients also carried a large gene deletion on one allele. However, the variants on the second allele were not perfectly matched as they presumably do not contribute to the tenascin-X haploinsufficiency or to the respective clinical symptoms, and interpretation is constrained by the small sample size. Interestingly, however, all CAH-X patients had a salt-wasting phenotype with another classic variant on the second allele without interfering with the TNXB gene, whereas the controls also had milder variants on the second allele, leading to a milder clinical CAH phenotype.

### 3.2 Serum TNXB protein concentrations do not correlate with clinical symptoms observed in our cohort

**Figure 2:**
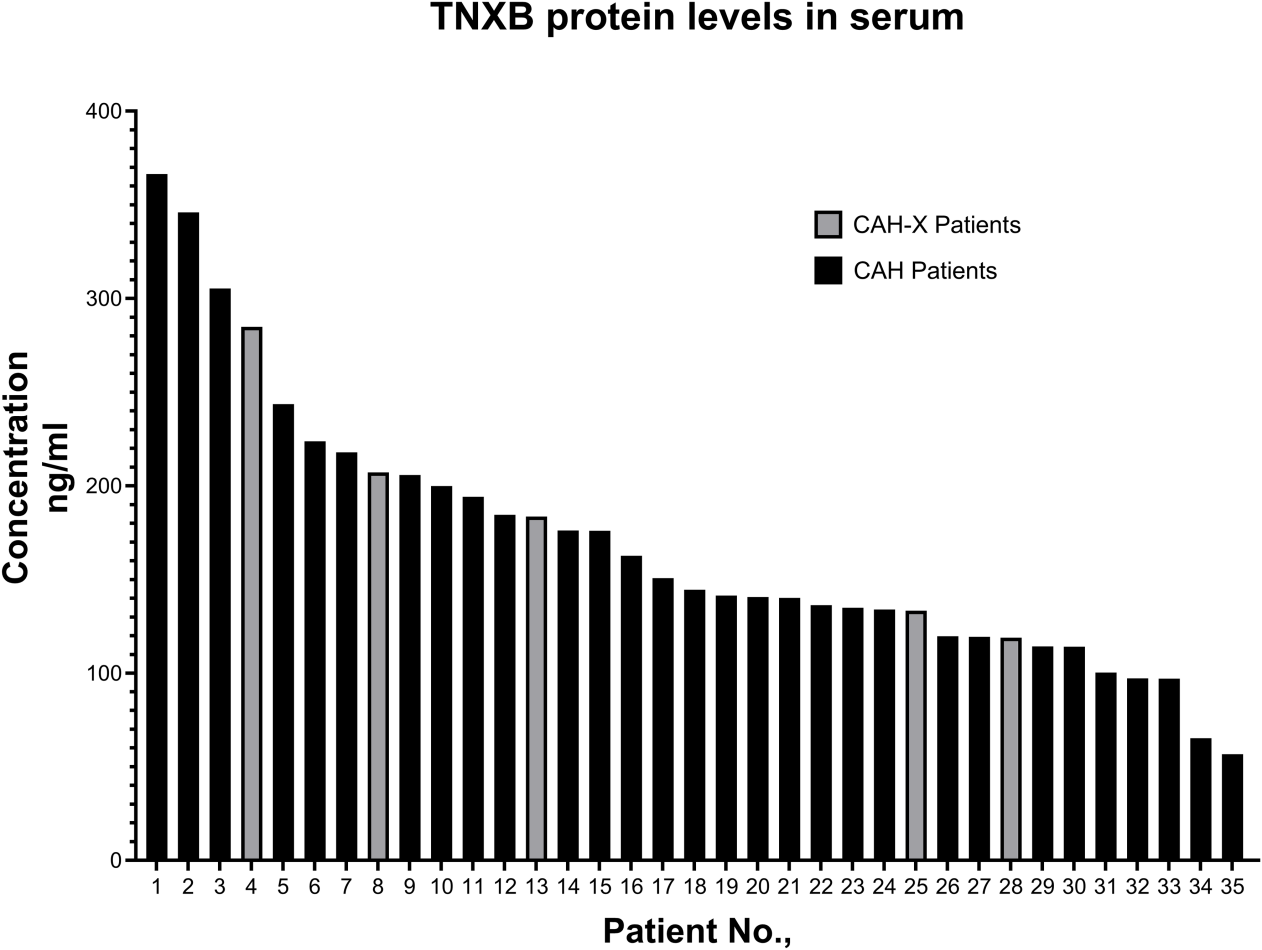
TNXB protein levels in the serum of CAH-X and CAH patients. TNXB protein levels in patient blood serum was measured by performing ELISA on 5 CAH-X subjects and 30 random CAH subjects. The graph represents the total sTNXB protein concentration (ng/mL) measured in different patient samples.

In order to investigate the correlation between serum TNXB protein levels (sTNXB) and clinical observations, tenascin-X levels in the blood serum of our patients were measured. In total, 35 samples including five patient samples that tested positive for the presence of CAH-X-CH1 chimera were analyzed for tenascin-X levels (**Figure 2**). Interestingly, sTNXB levels in CAH-X patients were elevated to a modest degree compared to the CAH control patients (average 185.56 vs. 166.92 ng/mL, **Figure 2**). Nevertheless, TNXB levels in both case and control groups were lower than the levels described in the average normal population (378 ng/mL) (26).

### 3.3 Assessing the clinical implications of the CAH-X CH1 chimera in our patient cohort

Four out of five CAH-X patients carrying the monoallelic *TNXA/TNXB* chimera and the CAH-X patient carrying the biallelic chimera (case subjects) were each matched with two CAH patients (control subjects) according to BMI and age (**Table 1**). Despite stringent measures taken to find adequate matching control CAH partners (control subjects) for five CAH-X patients (case subjects) similar in BMI, two case subjects had morbid obesity for which no matching partner within the control group of patients could be found. Therefore, partners with a slightly lower BMI were chosen (**Table 1**). In total, 15 subjects (6 men and 9 women) with a BMI between 17.6 and 41 kg/m^2^ and age between 24 and 54 years were categorized into five groups (group 1 to group 5, with the patient carrying the biallelic chimera and the corresponding control subjects corresponding to group 3) and examined for clinical symptoms associated with EDS.

**Table 1:** Bioelectrical impedance analysis of CAH-X and CAH patients in our study cohort.

Characteristic Features	Test group *n* = 5	Control group *n* = 10	Significance
Weight	87.20 ± 34.11	74.90 ± 12.74	*p* = 0.216
Height	165.20 ± 7.01	162.90 ± 7.46	*p* = 0.435
Age	39 ± 15	39 ± 15	NA
Sex (M/F)	2/3	4/6	NA
BMI (kg/m^2^)	31.44 ± 10.62	28.20 ± 4.49	*p* = 0.266
Body fat percentage	36.88 ± 10.26	30.16 ± 8.85	*p* = 0.257
Absolute fat mass	33.98 ± 19.75	23.10 ± 9.50	*p* = 0.339
Body water	38.96 ± 12.66	37.92 ± 5.93	*p* = 0.710
Lean muscle mass	53.22 ± 17.28	51.80 ± 8.09	*p* = 0.710
Extracellular matrix (ECM)	25.12 ± 7.10	23.44 ± 3.36	*p* = 0.346
Body composition monitor (BCM)	28.06 ± 10.25	28.34 ± 5.15	*p* = 0.902
ECM/BCM	0.922 ± 0.11	0.84 ± 0.09	*p* = 0.106
Cell percentage	52.14 ± 2.94	54.59 ± 2.66	*p* = 0.110

In order to perform clinical examinations with appropriate control subjects, each CAH-X patient was matched with two CAH patients based on both age and BMI. The case:control matching was made with SPSS using conditional logistic regression. The 15 participants were categorized into five groups (groups 1–5). The table represents an average comparison of both case (CAH-X) and control (CAH) groups.

Bioimpedance analysis showed no significant difference in body composition between the case and control subjects belonging to all five groups. An average BMI of 31.4 kg/m² was observed among the case subjects as opposed to the control subjects who had an average BMI of 28.2 kg/m² (**Table 1**). This relates to the incidence that, for two affected patients, no adequate BMI-matched partner could be found. Notably, 20% of the participants were affected in both the case and control groups. One of the case subjects and six of the control subjects indicated to be more physically active (sports >2 h/week). However, being physically more active did not significantly affect the overall lifestyle of the participants. Nevertheless, the control subjects from all five groups displayed a tendency to be physically more active than the respective case group. This is possibly reflected in the higher BMI observed in the case group. CAH-X patients tended to have a higher body fat content and more absolute body fat mass, without statistical significance.

None of the 15 participants belonging to the five groups in our study reported suffering from chronic pain in the questionnaire. Neither hernia nor vesicoureteral reflux was reported by the participants. No striking difference in frequency or occurrence of migraines, headaches, or gastrointestinal complaints was observed between the case and control subjects across the five groups. All case subjects reported suffering from both foot malposition and back pain at least once per month, and only four participants of the control group reported suffering from back pain and foot malposition (40%). However, the numbers are too small, and the difference is statistically insignificant (*p*-value = 0.31).

#### 3.3.1 Neurological and musculoskeletal assessment of our patient cohort

**Figure 3:**
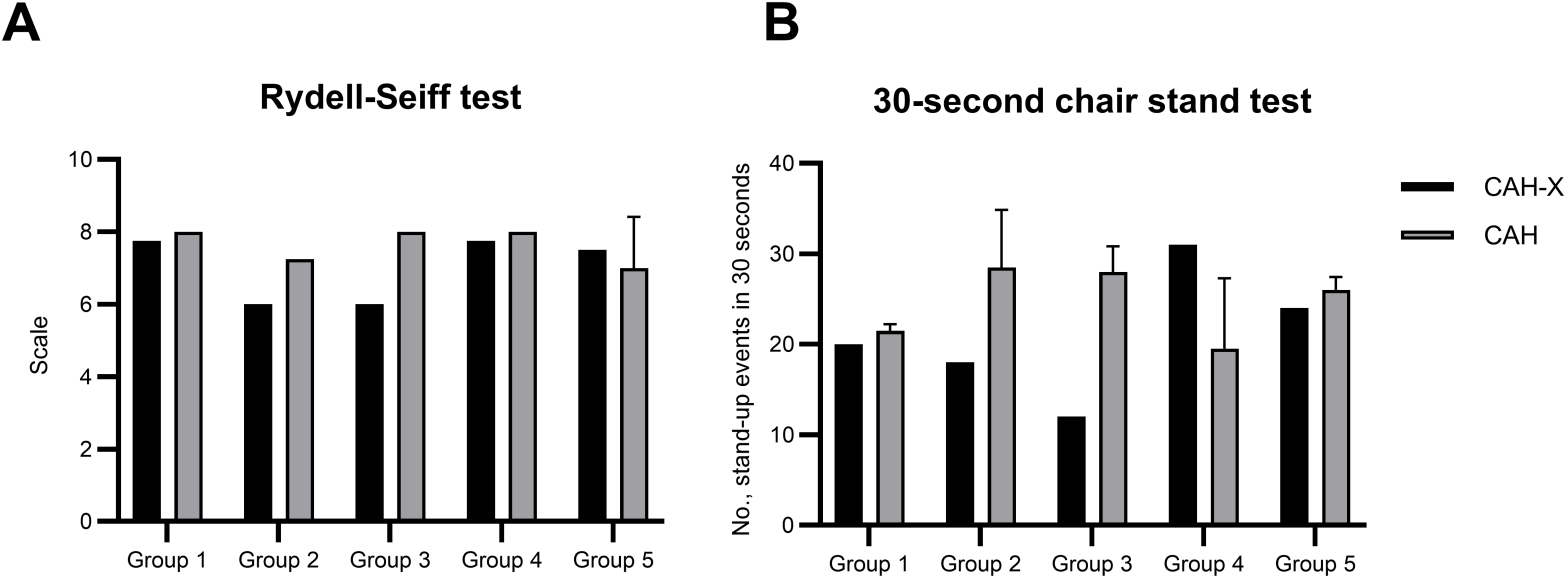
Neurological implications of the CAH-X CH1 chimera in our study cohort. **(A)** Proper functioning of the sensory and motor pathways was assessed by performing the Rydel–Seiffer turning fork test. A 128-Hz tuning fork was used on the participant’s inner ankle and wrist. The intersect position on the tuning fork is recorded the moment the participant no longer senses vibrations. The graph represents the average of score obtained from both the inner ankle and wrist. **(B)** A 30-s chair stand test was performed on the participants. The graph represents the number of times each participant could sit and stand in 30 s.

The Rydel–Seiffer turning fork test was performed to assess the proper functioning of the sensory and motor pathways. The assay was performed on the participant’s inner ankle and wrist. All participants displayed a ‘normal vibration sense’ (score 6–8) with the exception of the CAH-X patient carrying the biallelic chimera (group 3), who displayed a ‘mild sensory impairment’ (score 4–5) in the lower extremities (**Figure 3A**). CAH-X patients belonging to groups 2 and 3 performed less efficiently than the corresponding CAH control subjects in the 30-s chair stand test (**Figure 3B**). A similar trend was observed in CAH-X patients belonging to groups 1 and 5, although thisdid not reach statistical significance. Interestingly, the CAH-X patient belonging to group 4 performed slightly better than the corresponding control subjects. Observations from clinical tests including the Romberg, finger-nose, and heel-to-knee-to-toe tests were unremarkable. Pain sensitivity assessment did not yield any significant abnormality in the case subjects except for the CAH-X patient within group 1 who had a significantly higher pressure pain threshold than the CAH control subjects (**Supplementary Table 1**). Observations from reflex tests displayed a mild impairment in neuromuscular function among CAH-X patients. The difference is, however, statistically insignificant. CAH-X patients belonging to groups 1 and 3 displayed a higher pressure pain threshold than the respective CAH control patients, particularly in the tibia, deltoids, and thighs. However, the differences are statistically insignificant.

**Figure 4:**
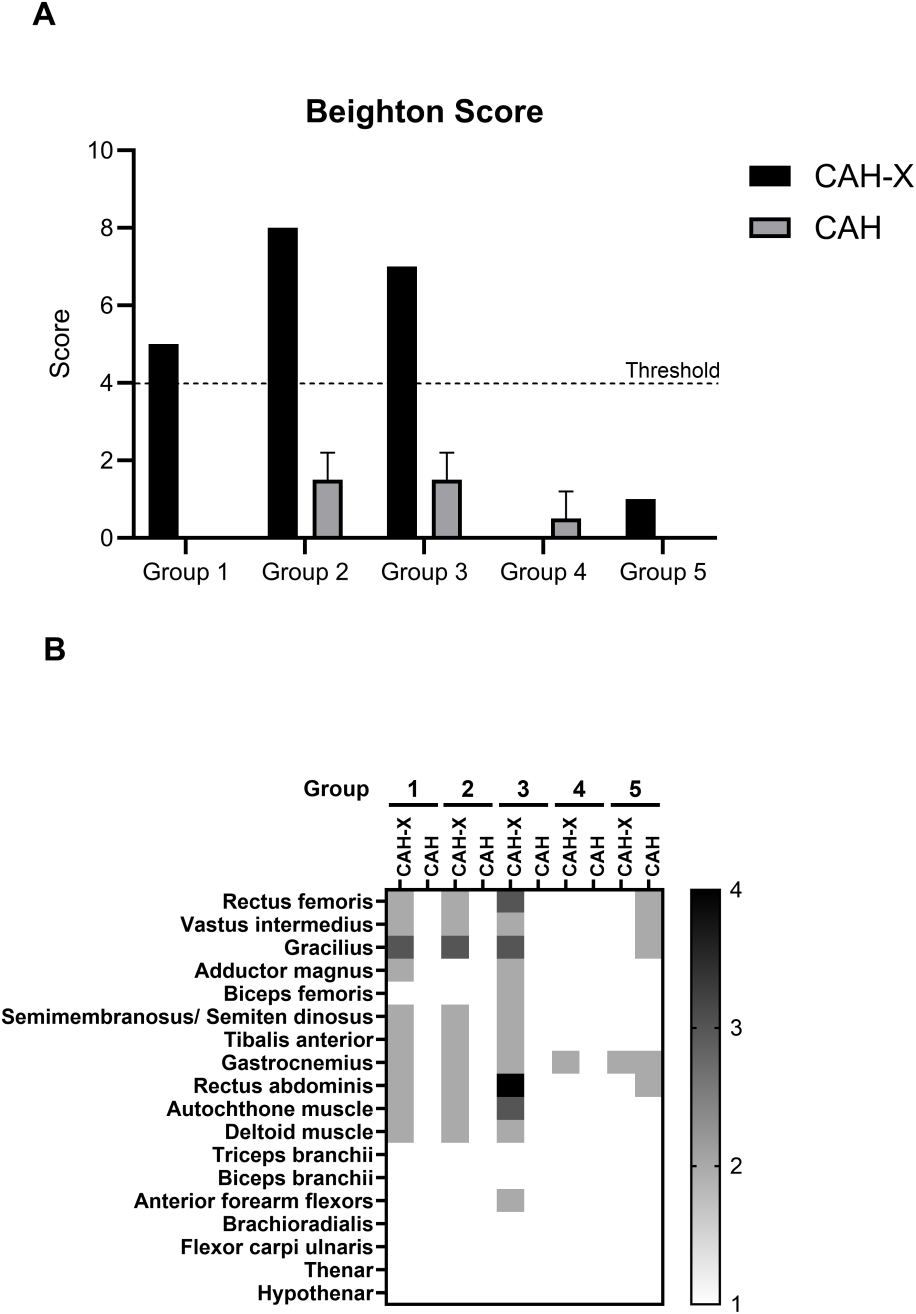
Musculoskeletal implications of the CAH-X CH1 chimera in our CAH study cohort. **(A)** The Beighton 9-point scale was used to examine participants for their ability to i) passively dorsiflex the fifth metacarpophalangeal joint beyond 90°, ii) oppose their thumb to the volar aspect of the ipsilateral forearm, iii) extend their knees beyond 10°, iv) extend their elbow beyond 10°, and v) bend forward and place the palms of their hands on the floor without bending the knee. For each positive result, 1 point was given, with 9 being the total score. The Beighton scale measurements for the participants in to groups 1 to 5 is represented here. A threshold of 4 is set and a score above 4 is considered a positive Beighton score. **(B)** Muscle echogenicity was assessed by performing muscle ultrasound examinations on different muscle groups in CAH-X patients and one corresponding CAH in groups 1–5. The muscles examined were the quadriceps, adductors, gastrocnemius, deltoids, finger extensors, median nerve, back, and abdominal muscles. The heatmap summarizes the modified Heckmatt scale used to classify the observed echogenicity/phenotype for the individual muscle groups examined. The lowest (grade 1) refers to normal muscle echogenicity (white), and the highest (grade 4) refers to a stronger increase in muscle echogenicity with complete loss of bone reflection (black).

CAH-X patients with higher sTNXB levels belonging to groups 1, 2, and 3 displayed a positive Beighton score. The patient carrying the biallelic *TNXA/TNXB* chimera (group 3) had a score of 7, while CAH-X patients belonging to groups 1 and 2 had scores of 5 and 8, respectively. No participant in the control group scored more than two points. Interestingly, the CAH-X patient belonging to group 4 performed better than the corresponding CAH control patients (**Figure 4A**). Additionally, all patients exhibiting a positive Beighton score also indicated back pain and pain in at least one joint.

Examination of strength in the dominant arm using a dynamometer showed no significant difference(*p* = 0.17). The CAH control participants seemed to be slightly stronger than the respective CAH-X patients. The MRC scale showed development of full strength in all participants. The FI-2 upper extremity test used to compare endurance and performance showed no significant difference (**Supplementary Table 1**).

Muscle ultrasound examinations performed on different muscle groups in CAH-X patients with higher Beighton scores (groups 1, 2, and 3) exhibited increased muscle echogenicity in most examined muscle groups and a Heckmatt scale of grade 3 in at least one muscle group—the gracilis muscle. Besides the gracilis muscle, the CAH-X patient belonging to group 3 also exhibited a grade 3 Heckmatt scale in the rectus femoris and autochthone muscle groups and grade 4 in the rectus abdominus muscle. CAH-X patients belonging to all groups exhibited an overall increase in muscle echogenicity with a grade 2 Heckmatt scale in most muscle groups, more specifically in the lower extremities, abdomen, and legs. None of the control CAH patients (belonging to the salt-wasting form of CAH) exhibited any muscle abnormalities, and all the examined control subjects displayed a grade 1 Heckmatt scale in all the examined muscle groups (**Figure 4B**).

#### 3.3.2 Dermatological assessment reveals a reduction in skin thickness in CAH-X patients

**Figure 5:**
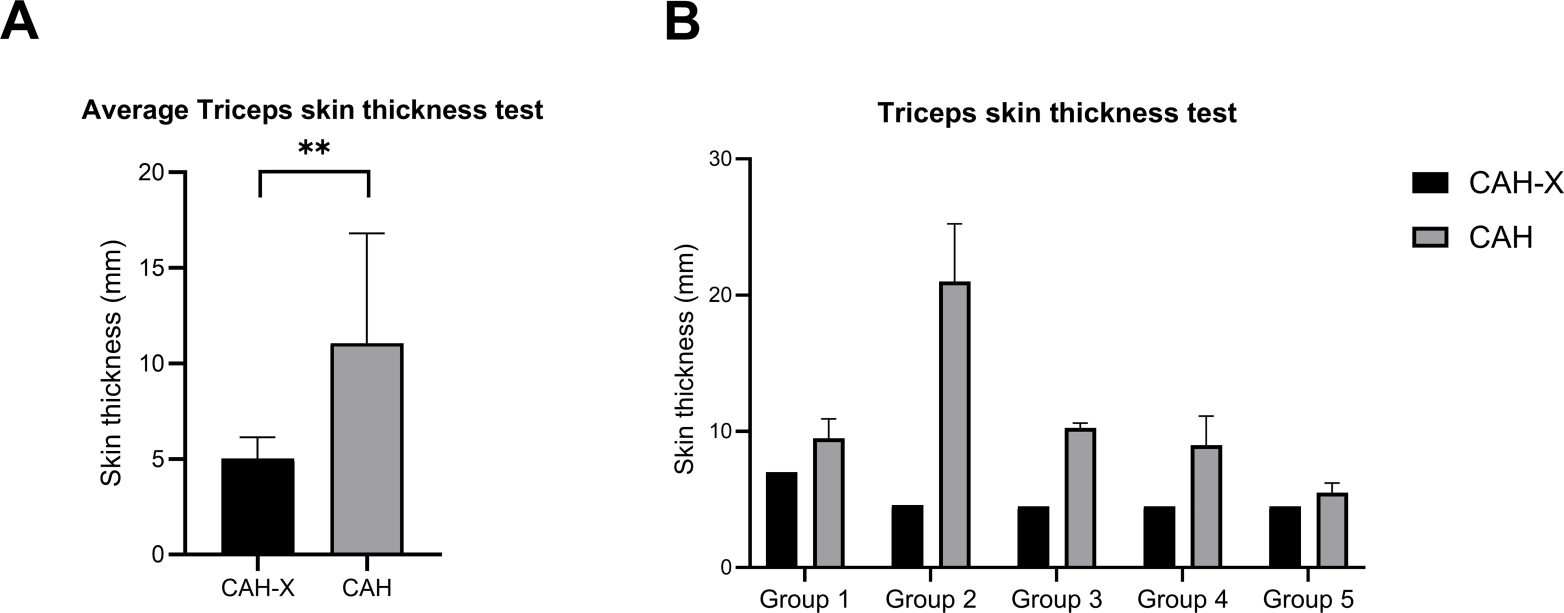
Dermatological implications of the CAH-X CH1 chimera in our study cohort. Skin thickness under the triceps was measured using a skin caliper. **(A)** Average skin thickness (mm) measured in both case and control subjects belonging to groups 1 to 5 is represented here (unpaired *t*-test, *p*-value < 0.05). **(B)** Skin thickness (mm) for the individual muscle groups examined. The lowest (grade 1) refers to normal muscle echogenicity, and the highest (grade 4) refers to a stronger increase in muscle echogenicity with complete loss of bone reflection. ** denotes statistical significance of p-value<0.05.

Most case and control subjects reported skin striations in the questionnaire; however, the CAH-X patient from group 3 reported suffering from them. Additionally, no striking difference in a subjective view of bruising themselves easily was observed between the two groups. Notably, 20% of both CAH-X and CAH control subjects reported frequently bruising themselves. Skin laxity was present in the patient with Ehlers–Danlos syndrome. Skin thickness at the triceps was measured, and patients with CAH-X in general had a lower skin thickness (average thickness ~5 mm) than the control group (average thickness ~11.05 mm), with a *p*-value of 0.0062 (**Figures 5A,B****;** **Table 2**).

**Table 2:** Skin abnormalities.

Participants		Injury frequency	Scarring	Striae
Group 1	CAH-X	0	0	1
	CAH	1	0	1
		0	0	0
Group 2	CAH-X	0	1	2
	CAH	0	0	2
		1	0	2
Group 3	CAH-X	1	0	1
	CAH	0	0	2
		0	0	1
Group 4	CAH-X	0	1	0
	CAH	0	0	1
		0	0	0
Group 5	CAH-X	0	0	0
	CAH	0	0	0
		0	0	0

Patients were asked to fill a questionnaire querying their frequency of skin injury, skin scarring, and striae formation. They were asked to grade their observations as follows: 0 = none (no frequent skin injury, no skin scarring, or no striae formation), 1 = low/low frequency, and 2 = high/high frequency of skin injury, skin scarring, and striae formation. 0 = no effect, 1 = mild effect, 2 = strong effect.

#### 3.3.3 Cardiovascular assessment reveals striking cardiac abnormalities in CAH-X patients in our cohort not previously associated with CAH

**Figure 6:**
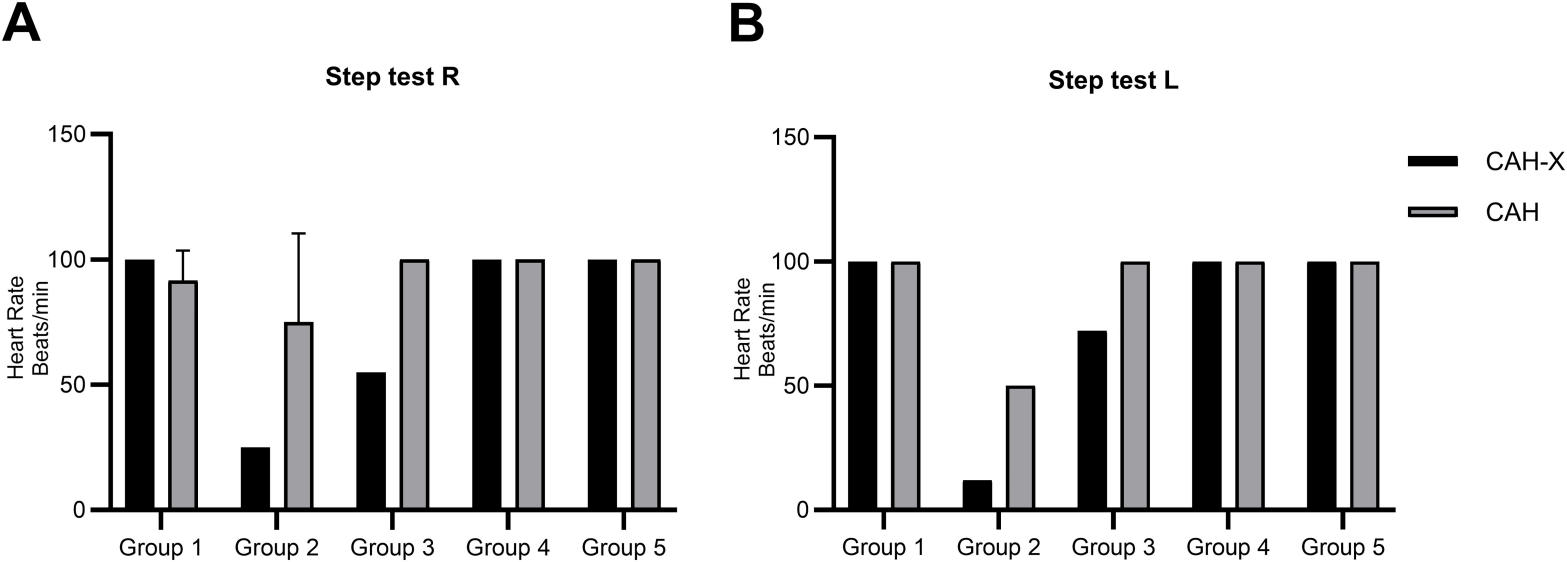
Cardiorespiratory fitness assessment of the CAH-X CH1 chimera in our study cohort. Cardiorespiratory fitness was assessed in patients by performing step tests. Participants were asked to step on and off a platform of a specific height either using their Right **(A)** or Left leg **(B)**, at a set pace for a period of 5 min, after which heart rate (HR) was measured during recovery. The normal heart rate is between 60 and 100 beats per minute.

CAH-X subjects belonging to groups 2 and 3 performed less efficiently than the respective CAH control subjects in cardiovascular fitness examinations (**Figure 6**). This reduced performance was observed in the step tests performed on both the right and left sides. Interestingly, CAH-X patients belonging to groups 1, 4, and 5 performed with the same efficiency on both sides as the corresponding CAH control subjects. The results of transthoracic examination were inconspicuous in two CAH-X patients belonging to groups 1 and 5. One CAH-X patient (group 4) had persistent truncus arteriosus that was surgically corrected at 6 months of age. A relaxation disorder was detected in the CAH-X patient belonging to group 3 and a minor mitral regurgitation in another (group 3). Interestingly, cardiac abnormalities previously described in CAH-X patients, such as mild valve regurgitation (groups 2, 3, 4) and dilation of the aortic root (group 4), were observed in our patient cohort. Nevertheless, taking into consideration the small group of patients in our study, no statistical abnormality or difference could be concluded for the screened parameters (**Table 3**).

**Table 3:** Cardiac abnormalities observed in CAH-X patients in our study cohort.

Parameters tested	CAH-X				
	Group 1	Group 2	Group 3	Group 4	Group 5
Chamber size • LV	Normal	Normal	Normal	Abnormal septal movement (post-OP)	Normal
• RV	Normal	Normal	Normal	Borderline function	Normal
• LA	Normal	Normal	Normal	Normal	Normal
• RA	Normal	Normal	Normal	Normal	Normal
Wall motion	Normal	Normal	Relaxation disorder	Normal	Normal
Valve function • Systolic function	Normal	Normal	Normal	Normal	Normal
• Aortic valve and root	Normal	Normal	Normal	Eccentric aortic insufficiency	Mild aortic insufficiency
• Tricuspid valve	Normal	Tightening without stenosis	Mild tricuspid insufficiency	Normal	Normal
• Mitral valve	Normal	Tightening without stenosis	Normal	Eccentric mitral insufficiency with thickened leaflet <5 mm	Normal
Pulmonary artery pressure	Normal	Mild mitral regurgitation	Mild tricuspid regurgitation	Mitral regurgitation	Normal
Pericardial effusion	No effusion	No effusion	No effusion	No effusion	No effusion

Echocardiography examination was performed on patients to assess chamber size, valve function, pericardium effusion, wall motion, and pulmonary artery pressure of the heart. The table summarizes the observations made on five CAH-X patients from groups 1 to 5.

## 4 Discussion

In our German cohort of CAH patients, fewer than 5% of CAH patients carry the CAH-X CH1 chimera. This is in stark contrast to the frequency rate estimated by independent groups from different geographic locations, ranging from 7% to 15%. Therefore, statistically significant differences or abnormalities could not be concluded in our examinations. Instead, our results and observations demonstrate trends between the CAH-X and CAH case subjects. Our study is the first to choose matching controls based not only on age and sex but also on BMI. In our study, no clinical observation made was age- or sex-dependent. Additionally, no striking influence of the CAH subtype was observed. In our study, serum tenascin-X (TNXB) protein levels in CAH patients were lower than the levels demonstrated in an average non-CAH population. Additionally, sTNXB levels were elevated in CAH-X patients compared to the corresponding CAH control subjects without 120 bp deletion to a modest degree, with the CAH-X patient from group 1 having the highest and the CAH-X patient from group 5 having the lowest amount of sTNXB protein levels.

Remarkably, CAH-X patients suffered from back pain and foot malposition and displayed a tendency toward mild neurological impairment; however, there was no statistical significance due to the small case study, except for the mean skin thickness test. Joint hypermobility (positive Beighton score) was observed in three CAH-X patients with high sTNXB levels (60%). Interestingly, groups 4 and 5, with low sTNXB levels, did not display a positive Beighton score. Three CAH-X patients (60%) exhibited increased muscle echogenicity. This corroborates the evidence that myopathy observed in CAH-X patients is due to an increase in phosphorylated SMAD1/5/8 (pSMAD1/5/8) resulting from abnormal bone morphogenetic protein (BMP) signaling (27, 28). Skin thickness was compromised in CAH-X patients among all five groups, with a statistical significance of <0.05 (*p* = 0.0062 using the unpaired *t*-test). Estimating tenascin-X levels in skin biopsies might provide further insight.

In our CAH-X patient cohort, previously described congenital ventricular diverticulum and quadricuspid aortic valve were not observed (29). Mitral valve abnormalities have been reported in 16% of *TNXB*-related clEDS patients within an English cohort (30). In our cohort, one CAH-X patient (20%) had mitral valve insufficiency. Two CAH-X patients had either persistent truncus arteriosus or diastolic dysfunction, which have not been previously described in *TNXB*-related clEDS. The patient with truncus arteriosus underwent surgical correction within 6 months after birth. During fetal development, an impairment of proper outflow tract (OFT) septation into the ascending aorta and pulmonary trunk results in persistent truncus arteriosus. Persistent truncus arteriosus observed in a mouse embryo deficient in α1 and β isoforms of retinoic acid receptors (RAR α1 and β) has been associated with increased TGFβ2 levels (31). Interestingly, CAH-X patients are reported to exhibit increased TGFβ2 levels. Whether increased TGFβ2 activation as a result of TNXB deficiency causes cardiac abnormalities observed in our cohort requires further molecular characterization of the mechanism.

In agreement with the study of Kolli et al. and Yamada et al., our study does not show a correlation between sTNXB levels and clinical observations in our cohort (32, 33). Whole exome sequencing (WES) or transcriptomic profiling of patient samples could provide insight into potential new players or novel *TNXB* missense variants. Independent studies have identified the *TNXB* loci to be differentially methylated resulting in low expression of *TNXB* in colorectal cancer cells. Interestingly, the *TNXB* promoter is differentially methylated in patients with psoriasis but without focal hypergranulosis, multiple sclerosis, and type 1 diabetes (T1D) (34–36). Whether TNXB protein levels in CAH-X patients are influenced by epigenetic reprogramming remains to be investigated. Moreover, as the *TNXB* gene is genomically embedded within the HLA loci, the influence of HLA haplotypes on *TNXB* gene expression cannot be excluded. Accordingly, HLA-DQB1*03 (one of the alleles found in T1D patients) has been associated with ~58.3% of the CAH-X CH1 chimeric allele, with a linkage disequilibrium pattern observed in 80% of probands with the CAH-X CH1 chimera (37).

Due to the novel cardiac and muscular abnormalities observed in our CAH-X patients, we advise regular cardiac and muscle ultrasounds to be performed on CAH patients with symptoms of joint hypermobility. Targeted physical therapy and exercises for joint stabilization should be implemented as a precautionary measure. Since our cohort is young on average, a longitudinal study must be conducted to understand the effect of mutation in older age.

## Data Availability

The original contributions presented in the study are included in the article/**Supplementary Material**. Further inquiries can be directed to the corresponding author/s.

## Supplementary material

Supplementary Table 1 Summary of clinical observations assessing muscular strength in participants. Table summarizes additional clinical observations made in our patient cohort. Muscle strength was assessed using MRC scale was used to assess muscle strength with 0 indicating no muscle strength and 5 indicating normal muscle strength, Quick motor function test (QMFT) score ranging from 0–80 and Dynamometer. Sensitivity to pain was measured using an Algometer on Tibia, Thighs, Deltoids and Brachioradialis muscles. Calf muscle strength and function was assessed by performing heel lift test. Tibialis anterior muscle strength was assessed using Toe-lift test.
