# Supplementary material for: Novel cardiac abnormalities observed in CAH patients with tenascin-X haploinsufficiency
Source: Front Endocrinol (Lausanne). 2026 May 15;17:1797669. doi: 10.3389/fendo.2026.1797669 (PMC13218977; doi:10.3389/fendo.2026.1797669)
Supplement: Supplementary Table 1 — Summary of clinical observations assessing muscular strength in participants. Table summarizes additional clinical observations made in our patient cohort. Muscle strength was assessed using MRC scale was used to assess muscle strength with 0 indicating no muscle strength and 5 indicating normal muscle strength, Quick motor function test (QMFT) score ranging from 0–80 and Dynamometer. Sensitivity to pain was measured using an Algometer on Tibia, Thighs, Deltoids and Brachioradialis muscles. Calf muscle strength and function was assessed by performing heel lift test. Tibialis anterior muscle strength was assessed using Toe-lift test. [file Table1.docx]

**Supplementary Table 1. Clinical observations from examinations performed to assess muscular strength in participants**

| **Parameters tested** | **Group 1** | | | **Group 2** | | | **Group 3** | | | **Group 4** | | | **Group 5** | | |
| --- | --- | --- | --- | --- | --- | --- | --- | --- | --- | --- | --- | --- | --- | --- | --- |
|  | **CAH-X** | **CAH** | | **CAH-X** | **CAH** | | **CAH-X** | **CAH** | | **CAH-X** | **CAH** | | **CAH-X** | **CAH** | |
| MRC | 5 | 5 | 5 | 5 | 5 | 5 | 5 | 5 | 5 | 4 | 5 | 5 | 5 | 5 | 5 |
| QMFT R/L | 79/80 | 80/80 | 80/80 | 74/80 | 78/80 | 76/80 | 71,5/80 | 80/80 | 80/80 | 80/80 | 80/80 | 80/80 | 80/80 | 79/80 | 80/80 |
| Dynamometer R/L (kg) | 48/38 | 46/40 | 47/43 | 38/36 | 34/30 | 38/34 | 24/23 | 30/28 | 25/22 | 20/22 | 34/35 | 32/24 | 32/38 | 40/38 | 42/41 |
| Algometer (kg/cm^2^) | | | | | | | | | | | | | | | |
| Tibia | >5 | >5 | 4 | 3 | 2.8 | 2.425 | 2.5 | >5 | 1.6 | 1.9 | >5 | 4.75 | >5 | 2.5 | >5 |
| Thighs | >5 | 4.6 | >5 | 2.75 | 3.5 | 2.65 | >5 | >5 | 3.4 | 3.2 | >5 | 3.5 | >5 | >5 | >5 |
| Deltoids | 4.75 | 3.4 | 4.75 | 1.5 | 2.1 | 1.025 | 1.5 | >5 | 2.25 | 1.5 | 4.525 | 2.75 | >5 | >5 | >5 |
| Brachoradialis muscle | >5 | 2.5 | 3.5 | 2.4 | 2.4 | 1.875 | 2.25 | 3.5 | 2.75 | 2 | 3.625 | 2.75 | 3.5 | 3.75 | >5 |
| Shoulder Reflux test R/L (%) | 100/100 | 75/52 | 100/100 | 58/38 | 83/50 | 67/83 | 100/83 | 100/100 | \| 100/100 \| \| --- \| | 53/70 | 100/100 | 83/100 | 100/100 | 100/100 | 100/100 |
| Shoulder Abduction test R/L (%) | 100/100 | 67/100 | 100/100 | 33/28 | 60/42 | 100/100 | 100/100 | 100/100 | 100/100 | 100/100 | 100/100 | 100/100 | 100/100 | 100/100 | 100/100 |
| Head raise test (%) | 100 | 67 | 66 | 23 | 58 | 40 | 50 | 57 | 100 | 67 | 100 | 83 | 100 | 100 | 100 |
| Hip Flexion test R/L (%) | 47/33 | 100/100 | 66/66 | 28/25 | 50/42 | 33/50 | 62/50 | 35/45 | 100/100 | 100/45 | 100/100 | 100/100 | 62/70 | 100/100 | 100/100 |
| Heel lift test (%) | 58 | 50 | 50 | 25 | 33 | 83 | 32 | 53 | 100 | 58 | 100 | 50 | 33 | 100 | 100 |
| Toe lift test (%) | 64 | 100 | 62.5 | 18 | 46 | 100 | 100 | 48 | 100 | 65 | 100 | 59 | 58 | 60 | 100 |

|  |
| --- |
|  |
|  |
|  |
|  |
|  |
|  |
|  |
|  |
|  |
|  |
|  |
|  |
|  |
|  |
